# Biocompatible Nutmeg Oil-Loaded Nanoemulsion as Phyto-Repellent

**DOI:** 10.3389/fphar.2020.00214

**Published:** 2020-03-17

**Authors:** Masturah Mohd Narawi, Hock Ing Chiu, Yoke Keong Yong, Nur Nadhirah Mohamad Zain, Muggundha Raoov Ramachandran, Chau Ling Tham, Siti Fatimah Samsurrijal, Vuanghao Lim

**Affiliations:** ^1^Integrative Medicine Cluster, Advanced Medical and Dental Institute, Universiti Sains Malaysia, Penang, Malaysia; ^2^Department of Human Anatomy, Faculty of Medicine and Health Sciences, Universiti Putra Malaysia, Serdang, Malaysia; ^3^Department of Chemistry, Faculty of Science, Universiti Malaya, Kuala Lumpur, Malaysia; ^4^Department of Biomedical Science, Faculty of Medicine and Health Sciences, Universiti Putra Malaysia, Serdang, Malaysia; ^5^Craniofacial and Biomaterial Sciences Cluster, Advanced Medical and Dental Institute, Universiti Sains Malaysia, Penang, Malaysia

**Keywords:** nutmeg oil, repellent activity, *in vitro* release, nanoemulsion, *Aedes aegypti*

## Abstract

Plant essential oils are widely used in perfumes and insect repellent products. However, due to the high volatility of the constituents in essential oils, their efficacy as a repellent product is less effective than that of synthetic compounds. Using a nanoemulsion as a carrier is one way to overcome this disadvantage of essential oils. Nutmeg oil-loaded nanoemulsion (NT) was prepared using a high speed homogenizer and sonicator with varying amounts of surfactant, glycerol, and distilled water. Using a phase diagram, different formulations were tested for their droplet size and insect repellent activity. The nanoemulsion containing 6.25% surfactant and 91.25% glycerol (NT 6) had the highest percentage of protection (87.81%) in terms of repellent activity among the formulations tested for the 8 h duration of the experiment. The droplet size of NT 6 was 217.4 nm, and its polydispersity index (PDI) was 0.248. The zeta potential value was –44.2 mV, and the viscosity was 2.49 Pa.s at pH 5.6. The *in vitro* release profile was 71.5%. When the cytotoxicity of NT 6 at 400 μg/mL was tested using the MTS assay, cell viability was 97.38%. Physical appearance and stability of the nanoemulsion improved with the addition of glycerol as a co-solvent. In summary, a nutmeg oil-loaded nanoemulsion was successfully formulated and its controlled release of the essential oil showed mosquito repellent activity, thus eliminating the disadvantages of essential oils.

## Introduction

Zika virus recently spread to Malaysia, with six cases reported in September 2016 (Subramaniam, 2016). Zika virus, which is a mosquito-borne flavivirus that is closely related to the dengue virus, first appeared in Brazil and then spread to countries in Latin America (Subramaniam, 2016). Other mosquito-borne diseases such as yellow fever, dengue, and chikungunya also have become major health problems in tropical and subtropical countries (Adeniran and Fabiyi, 2012; Gupta et al., 2013). Hence, the use of insect repellent products is essential to prevent these diseases (Rocha-Filho et al., 2014). N, N-diethyl-3-methylbenzamide (DEET) is the synthetic main active ingredient in commercial mosquito repellent products, and it has excellent repellency against a wide range of insects (Hang Chio and Yang, 2008). However, DEET poses health risks such as developmental toxicity in animals and environment (Iyer and Makris, 2010; Solomon et al., 2012).

With increased awareness of maintaining a green environment, biodegradable sources of mosquito repellent are widely promoted to replace chemical synthetic products that can have adverse effects on users (Maji and Hussain, 2009). Among available natural sources, essential oils from plants (Teng and Chen, 2017) are the preferred natural insect repellent because they contain rich sources of bioactive compounds, biodegrade to non-toxic products, and have less toxic on non-target organisms and the environment (Govindarajan, 2010; Lim et al., 2012; Assi et al., 2017). Plant essential oils are efficient insect repellents and provide direct protection to the user (Azeem et al., 2019). Although plant essential oils have been used in insect repellent products, their effectiveness is not significant (Bissinger and Roe, 2010). One possible reason could be that these products are characterized by uncontrolled release of the essential oil (such as, they either release too little or too much, with shorter duration of protection) (Solomon et al., 2012).

One potential way to solve the uncontrolled release problem is to encapsulate essential oils in nanoemulsions (Chen et al., 2020b). Preparation of a nanoemulsion involves mixing of two non-miscible liquids, such as oil and water, to produce spherical droplets with size ranging from 20 to 500 nm (San Martin-González et al., 2009). Their appearance is either transparent or slightly opaque (Sugumar et al., 2014). An additional constituent in nanoemulsions is surfactant, which acts as an emulsifying agent to decrease the interfacial tension between oil and water (Musa et al., 2013). Its presence prevents phase separation upon dispersion of the two immiscible liquids (Devarajan and Ravichandran, 2011). The stability of a nanoemulsion depends on its preparation. High energy emulsification provides intense disruptive forces to break up the mixture of oil, water, and surfactant into small droplets (Musa et al., 2013). Low energy emulsification involves the phase inversion method (Pant et al., 2014).

In this study, nutmeg oil-loaded nanoemulsion (NTs) systems were developed using glycerol, Montanov^®^ 82 (non-ionic surfactant), and deionized water. We aim to investigate the effect of high energy emulsification of the system consisting nutmeg oil, together with physicochemical characteristics, the release study and mosquito repellent activity of the nutmeg oil-loaded nanoemulsion.

## Materials and Methods

### Essential Oil and Standards

The essential oil, namely nutmeg oil (*Myristica fragrans*) was purchased from Imeltech Sdn. Bhd (Kuala Lumpur, Malaysia) with in-house code no. MF-004 (Batch no. NMO7956FT) and stored at the Herbarium Unit, Integrative Medicine Cluster, Advanced Medical and Dental Institute (IPPT). α-pinene (Batch no. SHBB7538V, Sigma-Aldrich, St Louis, United States), safrole (Batch no. BCBH2795V, Sigma-Aldrich, Shanghai, China), and terpinen-4-ol (Batch no. BCBF8537V, Sigma-Aldrich, Madrid, Spain) were used as standards for GCMS analysis.

### Animal and Test Organisms

Sprague Dawley (SD) rats were purchased from the Animal Research and Service Centre, Universiti Sains Malaysia (USM). Female *Aedes* (*Ae). aegypti* and DEET were supplied by the Vector Control Research Unit (VCRU), USM.

### Nutmeg Oil-Loaded Nanoemulsions

The essential oil-loaded nanoemulsion method was adapted from Sakulku et al. (2009) with slight modifications. Briefly, a phase diagram of surfactant, co-solvent, and water representing an apex triangle was plotted. Ternary mixtures with varying concentrations of surfactant (Montanov^®^ 82), co-solvent (glycerol), and double distilled water were prepared with a fixed nutmeg oil concentration (20% v/v). For each mixture, the total surfactant, co-solvent, and water summed to 100%. The mixture was emulsified using a high speed homogenizer (WT 130 hand-held homogenizer, Medigene, Selangor, Malaysia) at 18,000 rpm for 3 min. The formulation then was sonicated for 30 min using ultrasonicator cleaner (WUC-A10H, Wise Clean, Sonic Wise, California, United States) to obtain a submicron emulsion (Ragelle et al., 2012). Lastly, the formulation was centrifuged at 10,000 rpm for 15 min (Supermini Centrifuge, Hangzhou Allsheng Instruments CO., Ltd., Zhejiang Province, China). Visual observation was made immediately after centrifugation. Formulations with phase separation were rejected, whereas those without phase separation were chosen for particle size analysis. The desirable particle size was between 100 and 500 nm. The produced NTs were stored at 25°C until further use.

### Droplet Size and PDI

The particle size and PDI of the NTs were measured using the dynamic light scattering (DLS) technique at an angle of 173° and temperature of 25°C. This process was carried out using Zeta Nano-ZS apparatus (Malvern Instruments, Malvern, United Kingdom). Each NT sample was diluted with double distilled water until it reached the desired concentration, and all diluted samples were kept at a count rate of 150–300 k. Measurements were performed in triplicates.

### Mosquito Repellent Activity of NTs

#### Animal Ethics and Preparation of Animals

All animal procedures used were in strict accordance with animal care protocols, and all experimental protocols were approved by the Universiti Sains Malaysia Animal Ethics Committee [USM/Animal Ethics Approval/2013/(85) (441)]. SD rats (aged 8–10 weeks) were used for the insect repellent tests. The rats were kept at the Animal Research Facility, IPPT, USM under standard laboratory conditions for animals [temperature: 25 ± 2°C; relative humidity (RH): 55 ± 5%], with food and water provided and bedding replacement three times per week (Lim et al., 2012).

#### Test Organisms

The mosquitoes (*Ae. Aegypti*) were cultivated in the laboratory at constant temperature of 25–30°C and 80–90% RH at the VCRU, USM. A Whatman (Maidstone, United Kingdom) filter paper was placed in a Petri dish filled with chlorine-free water, which then was placed in an adult female mosquito’s cage. After eggs were laid, the Petri dish was removed and the eggs were allowed to hatch within 24 h. The newly hatched larvae were transferred into a rearing tray containing chlorine-free water and fed with a mixture of dog food, beef liver, yeast, and milk powder in the ratio of 2:1:1:1 w/w/w/w. Larvae were fed every day until pupation. At this stage, the pupae were separated from larvae and placed in a plastic cup with chlorine-free water in a mosquito cage. The plastic cup was removed from the cage when all adults had emerged. The adult mosquitoes were fed with vitamin B complex solution and 10% of sugar solution. Female adult mosquitoes of 5–7 days old were starved of blood for 24 h before being used in the repellent activity test.

#### Repellent Activity

The mosquito repellent tests of the selected NTs were conducted using female mosquitoes and rats as the blood donor. This method was adapted from Oshaghi et al. (2003) with slight modification. A rat was placed in a customized cage. The rat’s upper body (around 5 × 5 cm) was cleaned using ethanol and dried for 1–2 min before applying the NT. Approximately 1 mL of the NT was applied to the cleaned rat’s body. Another rat was treated with DEET (positive control), and another with liquid paraffin (negative control). Each rat was then placed in a mosquito cage containing 50 female *Ae. aegypti* mosquitoes for first 10 min of every hour for a total of 8 h. The number of mosquitoes that landed on the rat (10 s) within each 10 min interval was counted and compared to the controls. Each test was performed in triplicates with a new batch of mosquitoes. The numbers of landings in treated and control tests (positive and negative) were recorded, and the mean percentage protection from mosquito landing was calculated. The percentage protection is defined as the average number of bites received by the subject in each test and was calculated using equation (1):

(1) $$P\,e\,r\,c\,e\,n\,t\,a\,g\,e\,p\,r\,o\,t\,e\,c\,t\,i\,o\,n=\frac{\left(50-\text{NL}\right)}{50}\,\times 100$$

Where 50 = number of mosquitoes in the cage and NL = number of landings.

### Characterizations of NTs

#### Zeta Potential

The electrophoretic mobility and surface charge of each NT were measured by the frequency shift of scattered light at a 12° scattering angle using the Zeta Nano-ZS apparatus. Measurements were performed in triplicates.

#### Transmission Electron Microscope (TEM) Analysis

Visualization of the morphology and structure of the NTs was carried out by TEM analysis (Ghozali et al., 2015). A drop (1 μL) of NT diluted with deionized water (1:200) was placed on a 300 mesh copper grid and left for 1 min. The excess liquid was drawn off with Whatman filter paper. The grid was kept inverted and a drop of 2% (w/w) phosphotungstic acid (PTA) was applied to the grid for 10 s to negatively stain the sample. Excess PTA was removed by absorption on a Whatman filter paper. The grid was analyzed using a TEM Philips CM12 with Docu version 3.2 image analysis (Lim et al., 2013; Ayub et al., 2019).

#### Viscosity and Flow Analysis

Viscosity measurement and flow analysis of the NTs were performed using a dynamic shear rheometer (Rheologica Viscotech, Tarragona, Spain). A 40 mm diameter of parallel plate geometry was attached to the instrument and calibrated with a gap width of 1 mm. Next, 1500 μL of the NT were pipetted onto the surface of the rheometer with the absence of bubbles. The rheometer was run with the shear rate ranging from 0.1 to 1000 s^–1^ for 5 min, and the rate then was reduced from 1000 to 0.1 s^–1^ over the course of 5 min.

#### pH

The pH values of the NTs were determined directly using a calibrated pH meter (LIDA 92 series, Eutech Instruments Pte Ltd., Ayer Rajah Crescent, Singapore) at room temperature. The analyses were performed in triplicates.

### Release Study

#### *In vitro* Release of Nutmeg Oil

The release study was conducted using Franz-type diffusion cells (Carbone et al., 2014). First, 1 mL of NT was loaded on a cellulose acetate membrane with 0.2 μm pore size and 25 mm diameter. Prior to sample loading, the membrane was soaked in isopropyl myristate for 1 h to mimic the lipophilic barrier of the stratum corneum. Next, the membrane was mounted on top of the cells with 25 mL of water-ethanol (50:50) in the receiving compartment. This was to allow the “sink” condition and sustain essential oil solubilization. This compartment was constantly stirred at 700 rpm at 32°C and equilibrated for 30 min before collecting the first sample. An aliquot of receptor medium (1 mL) was collected at 0 min, 30 min, and then every hour thereafter for 24 h during the study. At each sampling time point, the system was replenished with the same volume of fresh, preheated receptor medium. The collected samples were analyzed by gas chromatography-mass spectrometry (GC-MS) after extraction with hexane. This experiment was performed in triplicates. The essential oil release kinetics from NTs was investigated by fitting the release data into Higuchi’s model, which can be expressed as:

(2) $$Q\,t=k\,t^{0.5}$$

where *Qt* is the percent of essential oil released at a given time (*t*) and *k* is the release rate.

### GC-MS Analysis

The collected samples from the release study were extracted using the liquid–liquid extraction technique for GC-MS analysis (Donsì et al., 2012). First, 1 mL of sample was dissolved in 5 mL of n-hexane and vortexed vigorously for 1 min. Sodium sulfate was added to the separated organic phase to remove the residual water. The GC-MS method was adapted from Lim et al. (2019). For sample analysis, 1 μL of diluted extracted sample in n-hexane (1:1000) was auto injected in the splitless mode. The constituents in the essential oil were identified by comparison with the National Institute of Standard and Technology mass spectral library and presented as relative percentage of the total peak (Tan et al., 2014; Lim et al., 2015).

### Cytotoxicity Assay

#### Cell Preparation

L929 cells (mouse fibroblast cells) were obtained from the American Type Culture Collection (ATCC). The preheated (37°C) culture vessel and medium were placed in a biosafety cabinet class II (ESCO Labculture^®^, Esco Micro Pte Ltd., Changi South Street, Singapore). The morphology of the cells was checked using an inverted microscope (CKX41 SF, Olympus, Tokyo, Japan). L929 cells were seeded in 75 cm^2^ culture flasks that contained Dulbecco’s modified eagle medium supplemented with 15% phosphate buffered saline (PBS), 5 mL penicillin streptomycin, and 5 mL L-glutamine. Next, the medium was aspirated and cells were washed twice with 5 mL PBS. Trypsin (5 mL) was added to disaggregate the cells, which then were incubated in a CO_2_ incubator (Heraeus BB15, Thermo Scientific, Massachusetts, United States) at 37°C for 15 min. Then, 10 mL of warm medium were added to the cells after detachment to stop the trypsinization and to disperse the cells. The cells were centrifuged (Heraeus Megafuge 16 centrifuge, Thermo Scientific) at 1000 rpm for 5 min, and the supernatant was aspirated. Lastly, 5 mL of fresh warm medium was added and pipetted up and down with the pellet, then 50 μL of the cells were added to 50 μL of trypan blue to calculate the cell using hemocytometer (Yakop et al., 2018).

#### Sample Dilution

For cell treatment, selected NT formulations were diluted with deionized distilled water in concentrations ranging from 50 to 400 mg/mL. The same procedure was repeated for DEET.

#### 3-(4, 5-Dimethylthiazol-2-yl)-5-(3-Carboxymethoxyphenyl)-2-(4Sulfophenyl)-2H-Tetrazolium (MTS) Assay

L929 cells were seeded at 3 × 10^3^ cells/mL into 96-well plates containing 100 μL of medium (Hanan et al., 2018; Poobalan et al., 2018). Next, 50 μL of diluted NT and DEET at different concentrations were added to the cells. Cells were incubated for 48 h with untreated cells as the control. Each plate was then treated with 20 μL of MTS reagent, and absorbance was read at 490 nm after 4 h using a microplate reader (FLUOstar Omega, BMG LABTECH, Offenbury, Germany). Cell viability (%) was calculated using the following formula:

(3) $$Cellviability\left(\%\right)=\frac{O\,D\,s\,a\,m\,p\,l\,e}{O\,D\,c\,o\,n\,t\,r\,o\,l}\times 100$$

where *OD* is the absorbance from the microplate reader.

### Thermodynamic Stability Study

The preliminary stability of the NTs was evaluated at 24 h by centrifuging at 15,000 rpm for 15 min. Samples showing layer separation were eliminated. Samples without layer separation were stored at 25 ± 2°C, 60 ± 2°C, and 4 ± 2°C. Droplet size and pH were measured at 1, 30, 60, and 90 d. The experiment was performed in triplicates.

### Statistical Analysis

Values were expressed as mean ± standard deviation (SD). Differences among samples were examined using one-way analysis of variance. A difference was considered to be statistically significant at *p* < 0.05.

## Results and Discussion

### Phase Diagram for NTs

**FIGURE 1:**
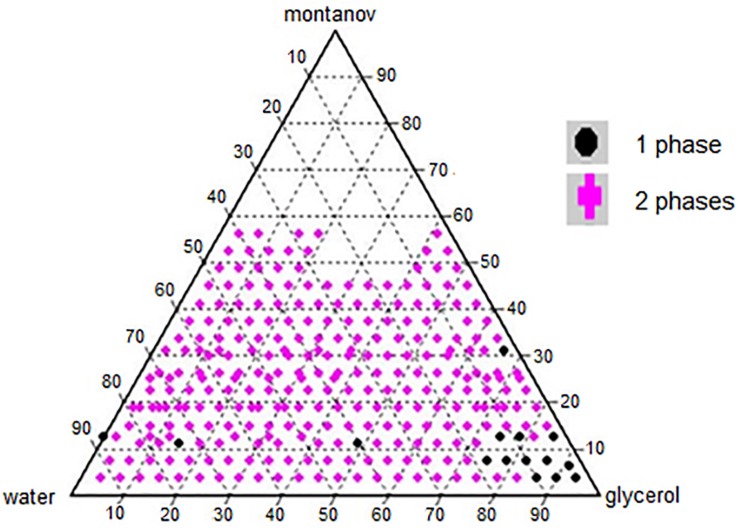
Ternary compositions of surfactant, co-solvent (glycerol), and double distilled water.

A phase diagram of ternary mixtures with surfactant (Montanov^®^ 82), co-solvent (glycerol), and double distilled water with nutmeg oil at a fixed concentration of 20% v/v was constructed (Figure 1). Each apex on the triangle representing the ternary system represents 100% of the component at that apex. The amount of surfactant used in the formulation ranged from 3.75 to 56.25%, that of glycerol ranged from 0.0 to 93.75%, and that of double distilled water ranged from 3.75 to 93.75%. In total, 296 formulations were constructed on the phase diagram. Visual observation was made immediately after the preparation of each formulation. Formulations that appeared as one phase were selected, and their average particle size was determined by the DLS technique using the Zeta Nano-ZS apparatus. Formulations with droplet size of ≤500 nm were selected as NTs for further testing (Pant et al., 2014).

Table 1 shows the composition of the single phase nanoemulsions in the ternary system. NT 13 contained the highest percentage of surfactant (31.25%) and NTs 11, 14, and 15 had the lowest (3.75%). The amount of co-solvent in these NTs ranged from 93.17% (NT 15) to 15% (NT 3). NT 1 was formulated with 12.5% surfactant and 87.50% water, without any co-solvent.

**TABLE 1:** Composition of NTs.

Sample	% (v/v) of surfactant	% (v/v) of	% (v/v) of
	(Montanov^®^ 82)	glycerol	H_2_O
NT 1	12.50	0.00	87.50
NT 2	12.50	75.00	12.50
NT 3	11.25	15.00	73.75
NT 4	11.25	48.75	40.00
NT 5	12.50	78.75	8.75
NT 6	6.25	91.25	2.50
NT 7	12.50	85.00	2.50
NT 8	7.50	75.00	17.50
NT 9	7.50	78.75	13.75
NT 10	7.50	82.50	10.00
NT 11	3.75	86.25	10.00
NT 12	7.50	86.25	6.25
NT 13	31.25	66.25	2.50
NT 14	3.75	90.00	6.25
NT 15	3.75	93.75	2.50

The construction of phase diagram is crucial to determine the single-phase boundary at each percentage of surfactant, co-solvent, and water with a fix percentage of nutmeg oil (20%) to yield a nanoemulsion in low free energy and thermodynamically spontaneous (Shakeel et al., 2008). The regions of the nanoemulsion in the phase diagram shown in Figure 1 were marked after visual observation of the formulated samples to identify those that were monophasic, clear and transparent mixtures after stirring, sonication, and thermodynamic stability testing (Devarajan and Ravichandran, 2011).

A surfactant or emulsifying agent is important in formulating a nanoemulsion because it reduces interfacial energy between oil and the aqueous phase via formation of a layer around the droplets of the nanoemulsion and it provides a mechanical barrier to coalescence (Kotta et al., 2014). Hence, surfactants added to a water-in-oil system (nutmeg oil, water, and glycerol) increase the aqueous phase concentration due to micelle solubilization. In our study, nutmeg oil was attached to the hydrophobic region of the emulsifier micelle and transferred to the aqueous phase (Donsì et al., 2012). At high concentration of surfactant, smaller droplets of NT are formed, which decreases the interfacial tension between nutmeg oil and the aqueous phase (Wang et al., 2007). Phase separation of a nanoemulsion might occur at low concentration of surfactant due to incomplete coverage of surfactant to oil and aqueous phase (Musa et al., 2013). Therefore, identifying the optimal surfactant concentration was important for solubilizing the oil with micelle solutions with the maximum amount of surfactant at 10% to the oil ratio (Fernandez et al., 2004). Yuan et al. (2008) reported that the combination of Tween 20-Tween 40 (10%) and Tween 60–80 (6%) reached a plateau for droplet size of the nanoemulsion. This is because of full coverage by the excessive surfactant in the nanoemulsion system (Yuan et al., 2008). Tang et al. (2012) reported that the minimum droplet size of a nanoemulsion occurred when 5–6% concentration of Cremophore EL was used; further addition of surfactant led to an increase in droplet size of the nanoemulsion (Tang et al., 2012). In our study, 7.5% was the maximum surfactant percentage that led to the formation of nano sized droplets with an acceptable PDI value. Once the nutmeg oil in the formulation has reached the saturation level (micelles), any addition of water and surfactant would not further dissolve. Excess free surfactant that is not adsorbed to the oil and aqueous interface would cause flocculation due to the increase of local osmotic pressure; this would cause the surfactants to “flow out” from the intervening liquid between the droplets, which in turn would lead to phase separation of the formulated samples (Jafari et al., 2007).

Glycerol acted as a co-solvent in this system to increase the viscosity of NTs and to decrease the droplet collision frequency (Chanasattru et al., 2007). Glycerol is added to the aqueous phase of a nanoemulsion to modify the bulk physicochemical properties of the formulated samples, such as density, viscosity, refractive index, interfacial tension, optimum curvature, and solubility of the surfactant in the aqueous phase (Saberi et al., 2013). In our study, a high concentration of glycerol led to the nano formation of NTs (Saberi et al., 2013).

### Droplet Size and PDI of Formulated NTs

**FIGURE 2:**
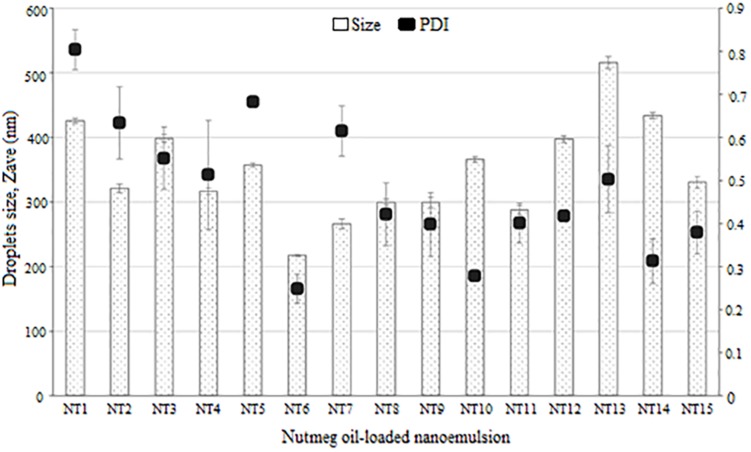
Droplet size and polydispersity index of NTs (mean ± SD, *n* = 3).

The 15 formulations with single phase from the phase diagram were selected to determine their droplet size and PDI values using the DLS technique and a Zeta Nano-ZS apparatus. DLS measures the z-average mean of the nanoemulsion (Yuan et al., 2008). Figure 2 shows the average droplet size and PDI of the selected samples. NT 6 exhibited the smallest droplet size (mean diameter 217.4 nm), and NT 13 had the largest droplet size (516.13 nm). As mentioned earlier, any NT with droplet size >500 nm was rejected. Therefore, NT 13 was eliminated from further analysis. Formulations with droplet size in the range of 200 to 300 nm were NT 7 (266.17 nm), NT 8 (299.83 nm), NT 9 (299.13 nm), and NT 11 (288.1 nm). The rest of the formulations had droplet sizes between 300 and 400 nm. The appearance of NT 6 was slightly transparent because of its tiny droplet size. Although some of the NTs appeared cloudy, their droplet size was still in the nano range. Salvia-Trujillo et al. (2015) also reported that the droplet size of their nanoemulsion was still in the nano range despite a slightly turbid physical appearance.

The DLS technique measures the Brownian motion of the droplets, which is referred as the intensity-weighted average hydrodynamic diameter of the emulsion. Large droplets move more slowly than small ones. Sufficient dispersion of the nanoemulsion is required to reduce the multiple scattering effect of Brownian motion (Leong et al., 2009). Small droplets have a greater surface area than large droplets, thus more surfactant is needed to cover this greater area (Yuan et al., 2008). For this reason, the droplet size of NTs decreased with increasing percentage of surfactant due to adsorption of high amounts of oil and water. This led to the increased surface area and reduced interfacial energy, which provided the mechanical barrier to coalescence (Carbone et al., 2014).

Polydispersity index is an important measurement of homogeneity and stability of a formulated nanoemulsion. A small value of PDI indicates homogeneity and a narrow distribution of the nanoemulsion system (Sugumar et al., 2014). PDI values range from 0 to 1 for width size distribution, where 0 indicates a monodispersed particle and >0.5 indicates a broad distribution (Borhade et al., 2012). The formulations with PDI values < 0.5 were NT 6 (0.248), NT 8 (0.421), NT 9 (0.398), NT 10 (0.278), NT 11 (0.401), NT 12 (0.418), NT 14 (0.313), and NT 15 (0.379). These eight formulations had low PDI values, which indicated they had a similar and narrow size distribution (stable and uniform) (Divsalar et al., 2012). Hence, they were chosen for use in the mosquito repellent test. In contrast, PDI values of NT 1, NT 2, NT 3, NT 4, NT 5, NT 7, and NT 13 were >than 0.5 and thus were eliminated from further study. High PDI values indicate an inhomogeneous and polydispersed distribution (Wissing and Müller, 2002). Polydispersity occurs when both large and small droplets are produced in a nanoemulsion system. When Ostwald ripening occurs, small droplets dissolve into the large droplets, leading to increased droplet size (Wissing and Müller, 2002). This system is unstable, and phase separation occurs in such formulations after 24 h.

### Repellent Activity of the Selected NTs

**FIGURE 3:**
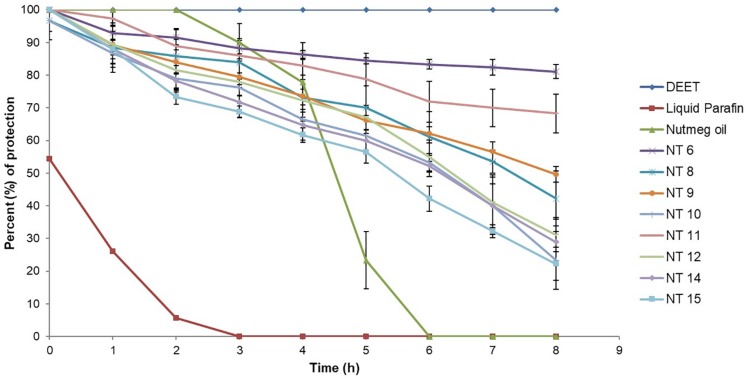
Mosquito repellent activity of controls, nutmeg oil, and selected NTs at hour intervals for the 8 h repellent test (Mean ± SD, *n* = 3).

Based on droplet size and PDI value, NT 6, NT 8, NT 9, NT 10, NT 11, NT 12, NT 14, and NT 15 were chosen for the mosquito repellent studies. These nanoemulsions did not show any phase separation after 24 h, indicating their stability. Figure 3 shows the mosquito repellent activity of the selected NTs at every hour interval throughout the 8 h repellent test. The mosquito repellent activities of these NTs were compared with those of nutmeg oil, DEET (positive control), and liquid paraffin (negative control). At hour 0, nutmeg oil and NT 6, NT 9, NT 11, NT 12, NT 14, and NT 15 provided full protection (100%) for the subjects; NT 8 and NT 10 provided 96.67% protection. Protection provided from hour 1 to hour 8 by NT 6 ranged from 92.94 to 81.09%, NT 8 (88.41 to 42.2%), NT 9 (89.35 to 49.57%), and NT 10 (86.67 to 23.33%). Protection provided by NT 11 and NT 12 ranged from 97.3 to 68.25% and 89.44 to 31.16%, respectively. NT 14 and NT 15 provided protection at 28.89% and 22.22% at hour 8, respectively.

**FIGURE 4:**
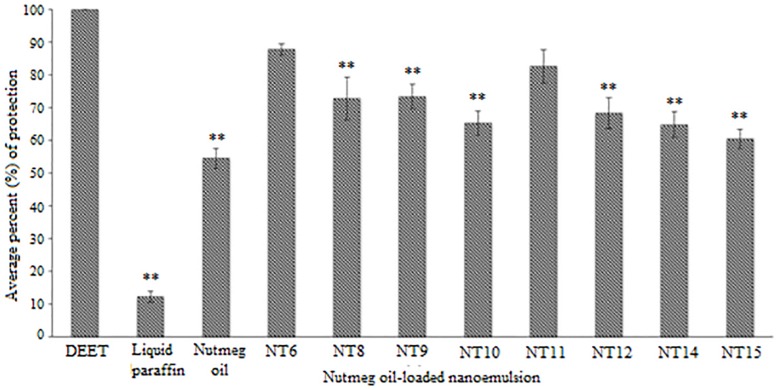
Average percentage protection against mosquitoes provided by controls, nutmeg oil, and selected NTs for 8 h (Mean ± SD, *n* = 3). ^∗∗^ *p* < 0.01 (Dunnett *t*-test).

Figure 4 shows the average percent protection provided by the NTs to the test subjects throughout the 8 h test. NT 6 exhibited the greatest repellency (87.81%) and NT 15 was least effective (60.54%). Other NTs were recorded at 72.77% (NT 8), 73.41% (NT 9), 65.31% (NT 10), 82.65% (NT 11), 68.36% (NT 12), and 64.86% (NT 14). The average percent protection by nutmeg oil was 54.57%. The protection provided by NT 6 and NT 11 did not differ significantly compared to that of the control (DEET), whereas the other NTs were significantly less effective than DEET (*p* < 0.01) (Rahman et al., 2015; Chong et al., 2018). In summary, NT 6 and NT 11 sustained their repellent activity every hour during the 8 h test. Therefore, these two NTs were selected for further characterization and the *in vitro* release study.

The selected formulated NTs exhibited higher percentage of protection compared to pure nutmeg oil. This proved that incorporating nutmeg oil into a nanoemulsion system could prolong the protection time. Although NT 6 and NT 11 could not provide greater protection than DEET (100% throughout the 8 h test), the toxicity of DEET should be taken into consideration. Natural essential oil-based repellent products are safe, pleasant to the user, and environmentally sustainable. The duration of protection against arthropod bites is important too, as essential oils contain an abundance of volatile compounds (Maia and Moore, 2011).

### Characterizations of NT 6 and NT 11

NT 6 and NT 11 were characterized for zeta potential, pH, viscosity, rheological behavior, and surface morphology. Table 2 shows the data for zeta potential, average pH, and viscosity of NT 6 and NT 11. The surface charge of a nanoemulsion is characterized by zeta potential, where values >+30 mV or<–30 mV lead to repulsive forces between the droplets (Sari et al., 2015). The zeta potential values were −44.2 and −62.9 mV for NT 6 and NT 11, respectively. These high values indicated good physical stability of these nanoemulsion systems (Nuchuchua et al., 2009). High repulsive forces between the droplets of a nanoemulsion prevent aggregation in the system and provide good physical stability (Chanasattru et al., 2007). The amount of surfactant present is important because electrostatic repulsion is required to provide the surface charge for stabilization (Musa et al., 2013). Musa et al. (2013) reported that the negativity of zeta potential decreased when the amount of surfactant (lecithin) present was reduced. This repulsive electrostatic interaction between the droplets tends to hinder coalescence, thereby increasing the surface area between the droplets (Musa et al., 2013). In fact, different nanoemulsion system exhibits different particle size distribution. Nanoemulsion system of citronella oil exhibited lower droplet size with increasing surfactant concentration (but with higher glycerol concentration) (Sakulku et al., 2009). The droplet size distribution significantly determines the delivery of the system in terms of rate and the duration of release (Chen et al., 2019a, b). In this study, the negative zeta potentials recorded for NT6 and NT11 were obtained because of the presence of the non-ionic surfactant Montanov^®^ 82.

**TABLE 2:** Zeta potential, average pH, and viscosity of NT 6 and NT 11 (*n* = 3).

Formulation	Zeta potential	pH	Viscosity (Pa.s) at stress
	(mV)	(mean ± SD)	stress 100 Pa (mean ± SD)
NT 6	−44.20	5.60 ± 0.16	2.49 ± 0.49
NT 11	−62.90	5.30 ± 0.13	0.09 ± 0.02

The pH values of NT 6 and NT 11 were 5.6 ± 0.16 and 5.3 ± 0.13, respectively. These pH values are suitable for topical administration to skin, as the pH of forearm skin is in the range of 4.2–5.9 (Borges et al., 2013). pH analysis is important to determine the stability and suitability of a product for topical application. In addition to pH, viscosity of a nanoemulsion is important for physical stability (Bali et al., 2010). Viscosities of NT 6 and NT 11 at stress of 100 Pa were 2.49 ± 0.49 and 0.09 ± 0.02 Pa.s, respectively. These results show that glycerol plays a major role in increasing the viscosity of the nanoemulsion, as glycerol constitutes a greater percentage of NT 6 compared to NT 11 (Sakulku et al., 2009). In addition, the viscosity of NT 11 was less than that of NT 6 due to the water content (NT 11 > NT 6). A higher surfactant ratio traps water in the cross-linking chain of the surfactant, which causes high viscosity of the nanoemulsion (Ghosh et al., 2013).

**FIGURE 5:**
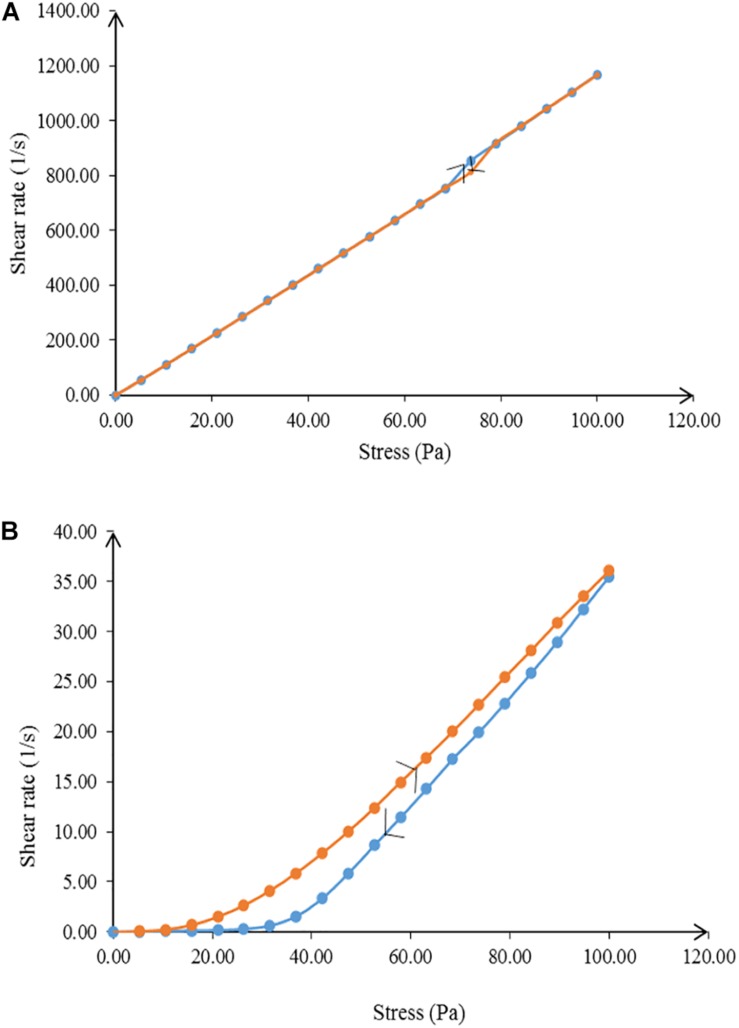
Flow behavior of **(A)** NT 6 and **(B)** NT 11 from 0.00 to 100 Pa and from 100 to 0.00 Pa shear stress.

Rheology, or flow behavior, is an important parameter for characterizing a nanoemulsion in terms of physical stability (Vasiljevic et al., 2006). The flow of a nanoemulsion affects the release and spreadability of the essential oil to the skin surface (Khurana et al., 2013). NT 6 exhibited non-Newtonian plastic behavior, whereas NT 11 showed Newtonian flow behavior (Figure 5) due to the difference in the water ratio. Varshosaz et al. (2013) stated that the coalescence of inner water droplets in the emulsion system gave effect on the behavior of the emulsion system. A nanoemulsion with low viscosity would not be adequate for an insect repellent product meant for topical skin application because it would tend to flow easily (Khurana et al., 2013). However, this problem was solved by the addition of glycerol as the co-solvent, which increased the viscosity of the formulation.

**FIGURE 6:**
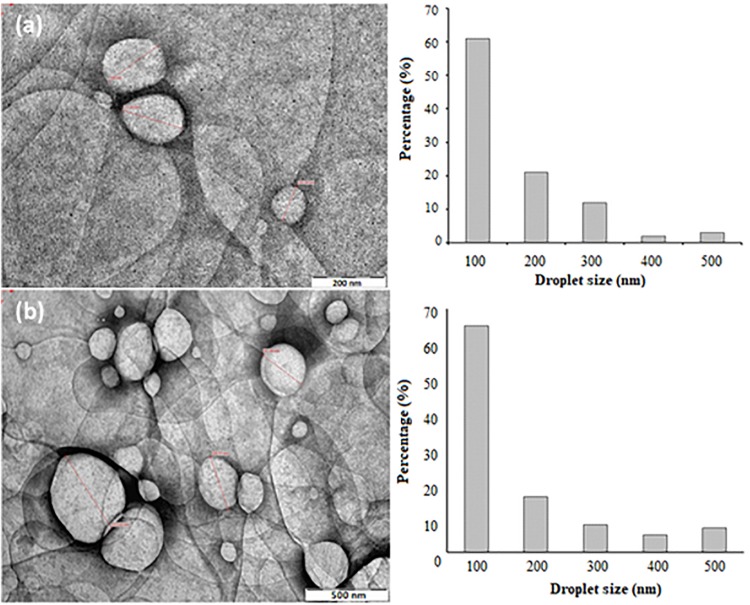
TEM morphologies and size distributions of **(a)** NT 6 at 66,000 magnifications **(b)** NT 11 at 40,000 magnifications.

Transmission electron microscope is another important way to analyze the surface morphology of NTs, as it provides a high resolution view of the *in situ* structure of the nanoemulsion (Bali et al., 2010). Figure 6 shows the surface morphologies of NT 6 and NT 11 (stained by PTA). NT 6 and NT 11 appeared bright with dark circles around the droplets, and the NTs showed both regular and irregular spherical shapes.

Droplet size of NT 6 was smaller than that of NT 11. The droplet size distribution in NT 6 and NT 11 ranged between 100 and 500 nm, and this correlated well with the results from the Zeta Nano-ZS apparatus (Figure 6). This correlation is important because the DLS measurement was performed via dilution of the nanoemulsion (Modi and Patel, 2011). The droplet size distribution of NT 6 and NT 11 showed the highest percentage at 100 nm (61% and 65%, respectively). Bali et al. (2010) also compared droplet size using two different approaches: TEM and photon correlation spectroscopy. TEM offers the advantage of providing high resolution for structures in the nano range, which cannot be detected by classical microscopy techniques (Salvia-Trujillo et al., 2013).

### *In vitro* Release of Nutmeg Oil From NT 6 and NT 11

**FIGURE 7:**
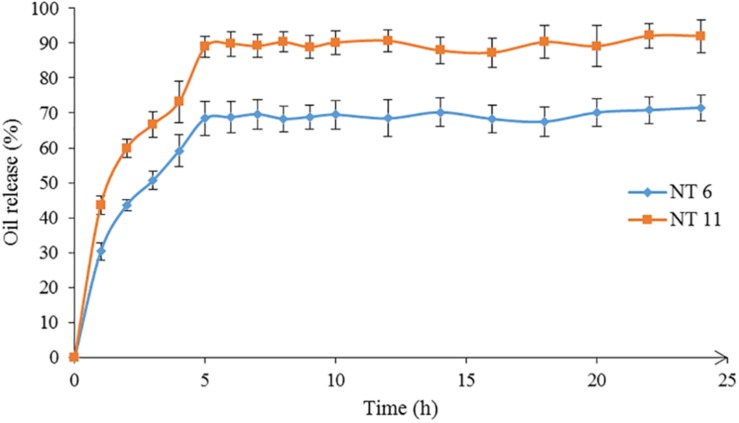
Release profiles of NT 6 and NT 11.

The *in vitro* release of nutmeg oil from NT 6 and NT 11 was investigated for 24 h (Figure 7). α-pinene (the major compound in nutmeg oil) was selected as the marker for this release study. The GCMS spectrum of the oil was published in Lim et al. (2019). Only 71.5% of nutmeg oil was released from NT 6 compared to 92.4% for NT 11. Release profiles of NT 6 and NT 11 fitted the Higuchi model and yielded *R*^2^ values of 0.9981 and 0.9866, respectively. NT 11 had higher flux and *k* value (release rate) than NT 6 (Table 3).

**TABLE 3:** *In vitro* release parameters of NT 6 and NT 11.

Formulation	J flux (mol dm^3^/cm^2^ s)	*R*^2^	*k* Value (cm^2^ s)
NT 6	0.00112	0.9981	1.0049 × 10^−5^
NT 11	0.00988	0.9866	7.8143 × 10^−4^

The release rate of an essential oil from a nanoemulsion is affected by viscosity, flow behavior, and water, surfactant, and co-solvent ratios in the nanoemulsion. NT 11 had a higher water ratio and exhibited higher flux and release rate compared to NT 6. Varshosaz et al. (2013) similarly reported that a metoprolol-loaded nanoemulsion (lecithin) with higher water ratio showed higher flux and release rate due to permeation enhancement of the desired compounds through cellulose acetate.

The slower release rate of NT 6 compared to NT 11 might be because NT 6 contained a higher percentage of surfactant (6.25%) compared to NT 11 (3.75%). Similarly, in a citronella oil-loaded nanoemulsion, the formulation containing 10% surfactant had a slower release rate compared to formulations with 2.5% and 5% surfactant (Sakulku et al., 2009). Therefore, surfactant content is an important factor affecting the release rate of an essential oil from a nanoemulsion system. In this study, the mobility of nutmeg oil and its release were easier at low surfactant percentage (i.e., NT 11). For NT 6, which contained a high level of glycerol, the release rate was lower because the glycerol caused the NT to be more viscous, which impacted the mobility of nutmeg oil in the carrier solution.

Despite its lower release rate, NT 6 showed greater mosquito repellent efficacy compared to NT 11. Sakulku et al. (2009) also reported that their citronella oil-loaded nanoemulsion with high percentage of glycerol and surfactant achieved a long duration of protection. These findings suggest that more viscous formulations prolong the protection time of repellent activity.

### Cytotoxicity of the NTs

Testing the safety of the NTs is important, as they are intended for use as a topical application for humans. The materials included in the formulation, such as nutmeg oil and surfactant, may cause some toxicity effect (due to the presence of monoterpenes in nutmeg oil) (Shakeel et al., 2008). Therefore, NT 6 and NT 11 were subjected to cytotoxicity analysis using L929 MTS assays.

**FIGURE 8:**
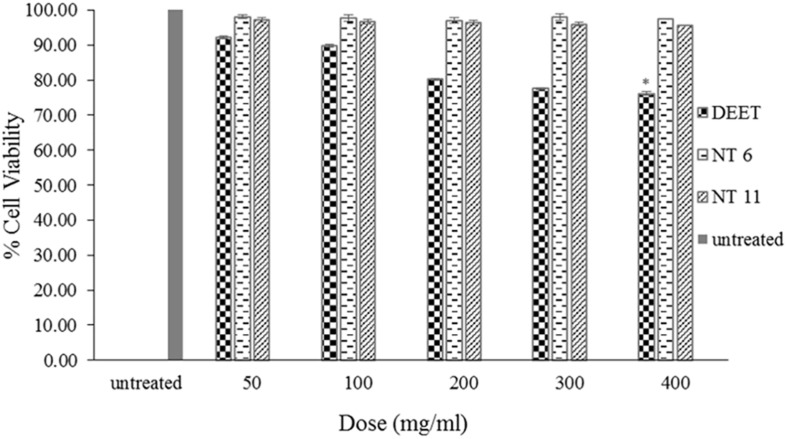
Percentage cell viability of L929 cells after 48 h exposure to NT 6, NT 11, and DEET at different doses of treatment using the MTS assay (mean ± SD, *n* = 3, ^∗^*p* < 0.05 (LSD test).

The cytotoxicity studies all were conducted using MTS assays (Chen et al., 2020a) with the same concentrations and cell lines (Figure 8). After 48 h of exposure to the formulations, the cell viabilities of the NT 6 group were 98.08% (50 mg/mL), 97.7% (100 mg/mL), 96.98% (200 mg/mL), 98.02% (300 mg/mL), and 97.38% (400 mg/mL). The NT 11 group exhibited cell viabilities at 97.1% (50 mg/mL), 96.81% (100 mg/mL), 96.36% (200 mg/mL), 96.02% (300 mg/mL), and 95.67% (400 mg/mL). The DEET group showed cell viabilities of 92.17% (50 mg/mL), 89.8% (100 mg/mL), 80.19% (200 mg/mL), 77.60% (300 mg/mL), and 76.16% (400 mg/mL), respectively. Comparison of results of the test compounds with those of L929 cell without treatment was made using the least significance difference (LSD) test. All samples showed no significance difference (*p* > 0.05), except for DEET at the highest dose (400 mg/mL) (*p* < 0.05).

These results indicate that NT 6 and NT 11 were not toxic to normal skin cells (Samad et al., 2018). In contrast, DEET at 400 mg/mL showed significant toxicity compared to the control. DEET is toxic to normal cell lines and causes skin eruption (Costanzo et al., 2007). The IC_50_ of the assay could not be determined because of low toxicity levels. Nerio et al. (2010) suggested that a low concentration of essential oil can reduce the toxicity and irritation effect. The amount of nutmeg oil (20%) used in the formulations in this study had no toxic effect on L929 skin fibroblast cells. Therefore, the formulated nanoemulsions appear to pose no risk of skin irritation or toxicity for topical application.

### Stability of NT 6 and NT 11

**FIGURE 9:**
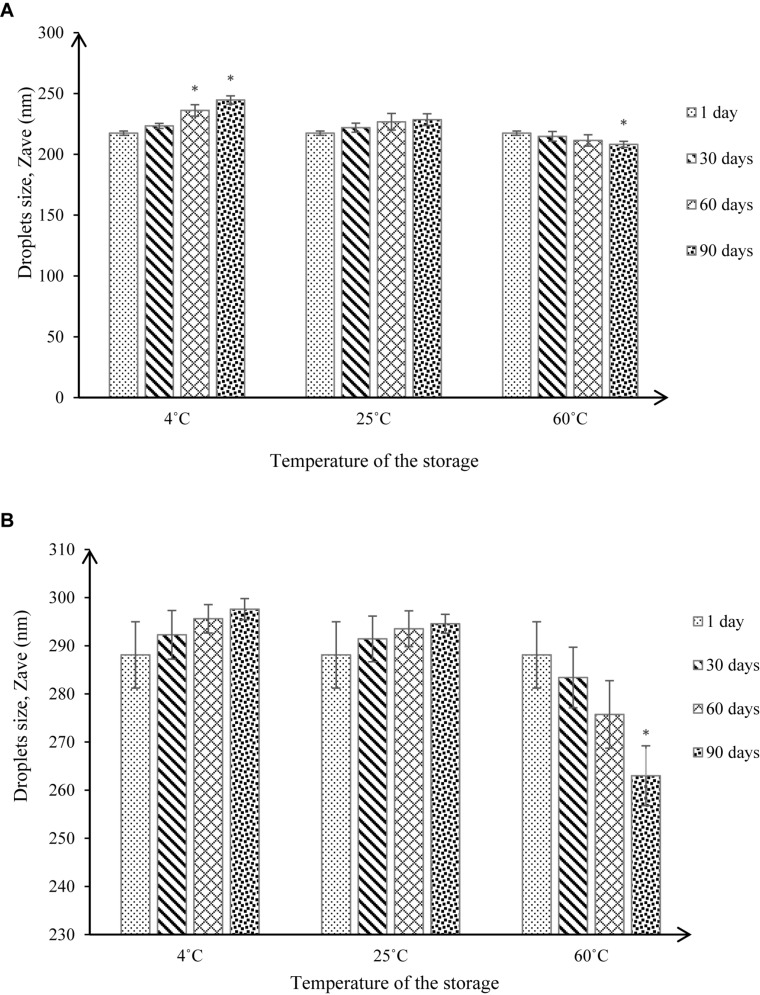
Droplet size under different storage conditions throughout 90-day stability tests for **(A)** NT 6 and **(B)** NT 11 (mean ± SD, *n* = 3, ^∗^*p* < 0.05).

The stabilities of NT 6 and NT 11 were measured based on droplet size and pH values at different storage temperatures (4°C, 25°C, and 60°C) and after 1, 30, 60, and 90 days of storage. Droplet size measurement (Figure 9) is a good indicator of stability, as a rapid increase of particle size indicates low stability (Bernardi et al., 2011).

At 4°C, the droplet size of NT 6 increased from 217.4 to 223.37 nm after 30 days of storage, showing no significant difference (*p* > 0.05 in droplet size increase compared to day 1). After 60 and 90 days of storage, the droplet size increased to 236.13 nm and 244.67 nm, respectively, which represented a significant difference (*p* < 0.05) compared to day 1 (217.4 nm). At 25°C, the droplet size of NT 6 kept increasing to 221.9 nm after 30 days, 226.73 nm after 60 days, and 228.53 nm after 90 days of storage. However, these values were not significantly different from the droplet size on day 1. In contrast, droplet size decreased from 214.77 nm (30 days) to 211.4 nm (60 days) and 208.17 nm (90 days) at storage temperature of 60°C. Only the value after 90 days of storage was significantly different from that on day 1 (*p* < 0.05).

For NT 11, droplet size at 4°C increased from day 1 (288.1 nm) to day 30 (292.27 nm), then to 295.6 nm and 297.6 nm after 60 and 90 days. The same trend occurred at 25°C of storage, where the droplet size increased from 291.43 nm (30 days) to 293.53 nm (60 days) to 294.51 nm (90 days). However, the increased values at both storage temperatures did not differ significantly from the droplet size at day 1 (*p* > 0.05). The droplet size at 60°C decreased from day 1 (288.1 nm) to day 60 (275.33), but the values did not differ significantly (*p* > 0.05). However, droplet size was 263 nm after 90, which was significantly smaller than droplet size on day 1 (*p* < 0.05).

NT 6 and NT 11 stored at 4°C exhibited turbidity, but the formulation was transparent when stored at high temperature (60°C). An anti-HIV nanoemulsion formulation also appeared turbid at low temperature but transparent after storage at room temperature; this was due to coagulation in the nanoemulsion system at low temperature (Kotta et al., 2014).

For NT 6 and NT 11, the increased droplet size at storage temperatures of 4°C and 25°C was still within the acceptable range for a nanoemulsion (i.e., ≤500 nm) (Pant et al., 2014), and phase separation was not observed throughout the storage period (Bernardi et al., 2011). These results indicate good stability in the nanoemulsion system. The significant increase in droplet size for NT 6 at storage temperature of 4°C after 60 and 90 days might be caused by Ostwald ripening, whereby small nanoemulsion droplets become larger. In addition, droplet aggregation by flocculation and coalescence between nanoemulsion droplets may occur (Rao and McClements, 2011).

**FIGURE 10:**
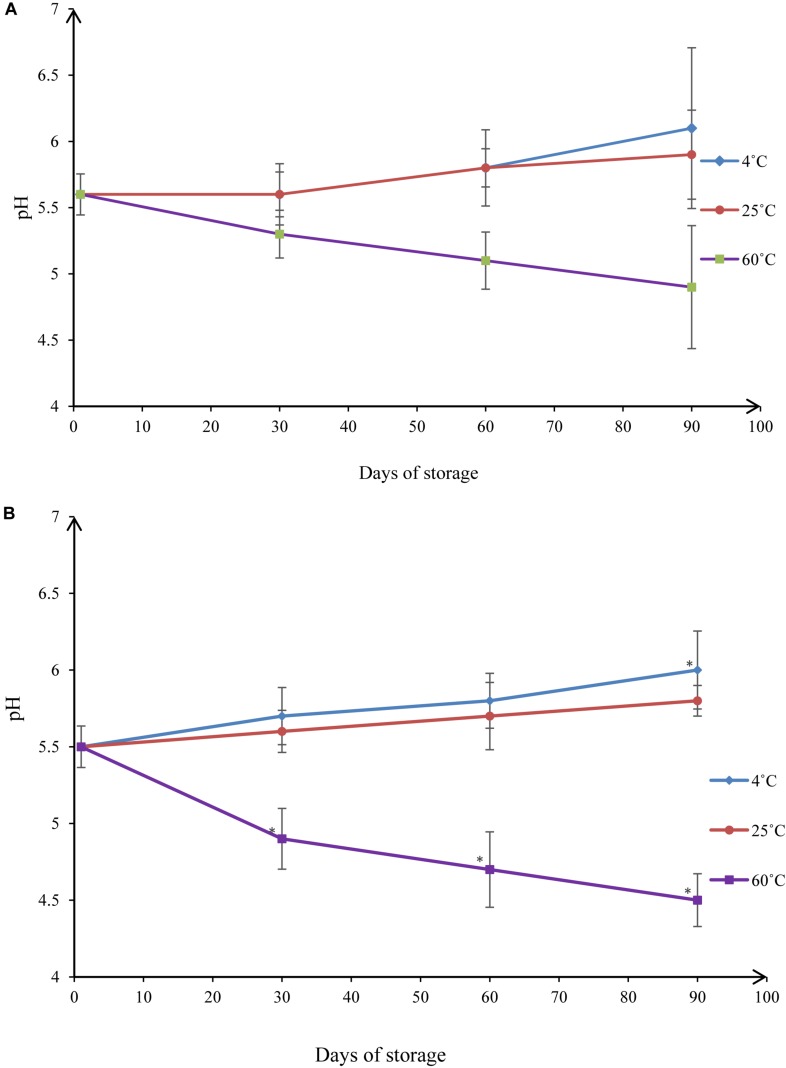
pH values under different storage conditions throughout 90-day stability tests for **(A)** NT 6 and **(B)** NT 11 (mean ± SD, *n* = 3, ^∗^*p* < 0.05).

pH monitoring is another way to assess the stability of a nanoemulsion (Figure 10). Changes in pH indicate a chemical reaction (Bernardi et al., 2011). pH values of NT 6 were 5.6 (day 1), 5.6 (day 30), 5.8 (day 60), and 6.1 (day 90) at 4°C. Values at 25°C were 5.6 (day 1), 5.6 (day 30), 5.8 (day 60), and 5.9 (day 90). These values did not differ significantly from those at day 1 (*p* > 0.05). At 60°C, pH decreased to 5.3 (day 30), 5.1 (day 60), and 4.9 (day 90). The pH value at day 90 differed significantly from that at day 1 (*p* < 0.05).

The pH values of NT 11 at 4°C storage temperatures were 5.5 (day 1), 5.7 (day 30), 5.8 (day 60), and 6.0 (day 90). At 25°C they were 5.5 (day 1), 5.6 (day 30), 5.7 (day 60), and 5.8 (day 90). For 4°C, the only significant difference detected was between the values at day 90 and day 1 (*p* < 0.05). No significant difference was detected between the control and any time point at 25°C (*p* > 0.05). At 60°C storage temperature, the pH decreased from 5.5 to 4.5 over the course of 90 days, and the values at days 30, 60, and 90 all differed significantly from the value at day 1 (*p* < 0.05).

These results show that the pH values of NT 6 and NT 11 were stable throughout 90 days of storage at temperatures of 4°C and 25°C. The range of reported pH values was acceptable for topical administration (5.4–6.9) (Borges et al., 2013). In summary, droplet size and pH of both NT 6 and NT 11 were stable at storage temperatures of 4°C and 25°C but not at 60°C. NT 6 and NT 11 are currently stored at the Herbarium Unit, Integrative Medicine Cluster, IPPT, USM with voucher numbers MF-004-NT6 and MF-004-NT11, respectively.

## Conclusion

Incorporating of essential oil using a nanoemulsion system can be used to control the release of the oil and prolong the insect repelling effect. In this study, nutmeg oil-loaded nanoemulsions were formulated and tested for their effectiveness as mosquito repellents.

Previous studies of nutmeg, basil, and peppermint oils and their combinations indicated that nutmeg oil was the best at providing protection against mosquitoes for SD rats. Therefore, nutmeg oil was used in this study to formulate essential oil-loaded nanoemulsions to develop an effective controlled release natural repellent formulation.

NT 6 and NT 11 formed desirable droplet sizes (<500 nm) with PDI values < 0.5. Zeta potentials of the NTs also were within the acceptable range (>–30 mV). pH values ranged from 4.2 to 5.9, which is suitable for topical application. Viscosities of these NTs were affected by the amount of water and glycerol in the formulation. Release rates of nutmeg oil for NT 6 and NT 11 were 1.0049 × 10^–5^cm^2^ s and 7.8143 × 10^–4^ cm^2^ s, respectively. The amount of surfactant used to produce NT 6 and NT 11 was less than 10%. The low percentage of surfactant used meets sensory, regulatory, and economic objectives. The preparation of NTs using a high speed stirring homogenizer and sonication tools produced stable nanoemulsion systems, which would be economically effective for larger scale production. In conclusion, NT 6 and NT 11 provided high mosquito repellent efficacy compared to nutmeg oil only and may prove to be useful alternatives for insect repellent products in the future.

## Data Availability Statement

All datasets generated for this study are included in the article/supplementary material.

## Ethics Statement

All animal procedures used were in strict accordance with animal care protocols, and all experimental protocols were approved by the Universiti Sains Malaysia Animal Ethics Committee [USM/Animal Ethics Approval/2013/(85) (441)].

## Author Contributions

All the authors were involved in the designing of the experiments. Additionally, MM, HC, and NM conducted the experiments. MR, SS, YY, and CT analyzed and interpreted the data. VL and MM wrote the manuscript. VL supervised the entire study.

## Conflict of Interest

The authors declare that the research was conducted in the absence of any commercial or financial relationships that could be construed as a potential conflict of interest.
